# Serum endotrophin, a type VI collagen cleavage product, is associated with increased mortality in chronic kidney disease

**DOI:** 10.1371/journal.pone.0175200

**Published:** 2017-04-12

**Authors:** Anthony Fenton, Mark D. Jesky, Charles J. Ferro, Jacob Sørensen, Morten A. Karsdal, Paul Cockwell, Federica Genovese

**Affiliations:** 1 Department of Renal Medicine, Queen Elizabeth Hospital, Birmingham, United Kingdom; 2 College of Medical and Dental Sciences, University of Birmingham, Birmingham, United Kingdom; 3 Nordic Bioscience A/S, Herlev Hovedgade, Herlev, Denmark; Hospital Universitario de la Princesa, SPAIN

## Abstract

**Background:**

Patients with chronic kidney disease (CKD) are at increased risk of end-stage renal disease (ESRD) and early mortality. The underlying pathophysiological processes are not entirely understood but may include dysregulation of extracellular matrix formation with accelerated systemic and renal fibrosis. We assessed the relationship between endotrophin (ETP), a marker of collagen type VI formation, and adverse outcomes in a cohort of patients with CKD.

**Methods:**

We measured serum ETP levels in 500 patients from the Renal Impairment in Secondary Care (RIISC) study, a prospective observational study of patients with high-risk CKD. Patients were followed up until death or progression to ESRD. Cox regression analysis was used to assess the relationship between ETP and risk of adverse outcomes.

**Results:**

During a median follow-up time of 37 months, 104 participants progressed to ESRD and 66 died. ETP level was significantly associated with progression to ESRD (HR 1.79 [95% CI 1.59–2.02] per 10 ng/mL increase; HR 11.05 [4.98–24.52] for highest vs lowest quartile; both *P*<0.0001). ETP level was also significantly associated with mortality (HR 1.60 [1.35–1.89] per 10 ng/mL increase; HR 12.14 [4.26–34.54] for highest vs lowest quartile; both *P*<0.0001). After adjustment for confounding variables, ETP was no longer significantly associated with progression to ESRD but remained independently associated with mortality (HR 1.51 [1.07–2.12] per 10 ng/mL increase, *P* = 0.019).

**Conclusions:**

Serum ETP level is independently associated with mortality in CKD. This study provides the basis for further exploratory work to establish whether collagen type VI formation is mechanistically involved in the increased mortality risk associated with CKD.

## Introduction

Patients with chronic kidney disease (CKD) are at increased risk of end-stage renal disease (ESRD) and early mortality [1]. There is growing evidence that dysregulation of extracellular matrix (ECM) formation is associated with accelerated systemic and renal fibrosis and modulation of inflammation [2]. However, there is limited information on the events that drive this process in human CKD.

Collagen type VI (Col6) forms a network of beaded microfilaments in the ECM of most connective tissues, where it interacts with other ECM molecules and provides structural support for cells. In addition to a mechanical role, Col6 has cytoprotective functions such as the inhibition of apoptosis and oxidative damage, and the regulation of cell differentiation and autophagy [3–5]. Following secretion of Col6 from its producing cell, the C-terminus of its α3 chain is cleaved off—this cleaved fragment is known as endotrophin (ETP, also termed Pro-C6), and represents a dynamic read-out of Col6 expression [6]. Col6, as one of the most abundant proteins in the glomerular ECM, is upregulated in renal disease [7–12] and we have recently found that high levels of serum ETP are associated with adverse outcomes in patients with kidney transplants, including allograft failure (unpublished data). However, it is not known whether Col6 is dysregulated in CKD and if dysregulation is associated with adverse clinical outcomes.

To address this, we measured serum ETP as a marker of Col6 expression in a well-characterised prospective CKD cohort. We assessed the relationship between ETP level and clinical outcomes of progression to ESRD and mortality, including adjustment for known risk factors for adverse outcomes in patients with CKD.

## Subjects and methods

### Population

Five-hundred participants from the Renal Impairment in Secondary Care (RIISC) Study (NCT01722383) were included in the study. The RIISC study is a prospective observational cohort study designed to identify determinants of adverse outcomes in CKD, and detailed methodology has previously been described [13].

In brief, patients in nephrology clinics in a single renal centre in Birmingham, UK, with non-dialysis high-risk CKD were invited to participate in the study. High risk CKD was as defined by the UK National Institute for Health and Care Excellence 2008 CKD guideline [14], and comprised one or more of: estimated glomerular filtration rate (eGFR) < 30 mL/min/1.73m^2^, or eGFR 30–59 mL/min/1.73m^2^ with a decline of ≥5 mL/min/1.73m^2^/year or ≥10 mL/min/1.73m^2^/5years, or a urinary albumin:creatinine ratio (ACR) ≥70 mg/mmol on three occasions. Patients were excluded if they had received immunosuppression for immune-mediated renal disease or if they were on renal replacement therapy (RRT). Patients consented to follow-up for 10 years, or until death or the initiation of RRT. Ethical approval was granted by South Birmingham Local Research Ethics Committee (reference: 10/H1207/6). All patients provided written consent, and the study was conducted in accordance with the Declaration of Helsinki.

All participants had demographic, clinical, and laboratory data collected at recruitment and during follow-up to produce detailed bio-clinical phenotyping. Comorbidity was recorded by specific comorbidity and by calculating each individual’s age-adjusted Charlson Comorbidity Index (CCI) [15]. Socioeconomic status (SES) was assessed using the Index of Multiple Deprivation (IMD 2010) [16]—an individual was assigned a score and rank according to their postcode; lower scores and ranks indicate greater deprivation. Body mass index (BMI) was calculated as body weight (kg) ÷ height (cm) squared. Blood pressure (BP) was measured using the BpTRU fully automated sphygmomanometer (BpTRU Medical Devices, Coquitlam, BC, Canada), which obtains a series of six BP readings at one-minute intervals following a five-minute rest period. Mean BP was derived from the average of the second to sixth BP reading, which has been reported to be comparable to mean daytime BP from 24-hour ambulatory BP monitoring [17]. Mean arterial pressure (MAP) was calculated using the average systolic (SBP) and diastolic blood pressure (DBP) and the following equation: *DBP* + (*SBP* − *DBP*] ÷ 3). Pulse pressure (PP) was calculated as *SBP* − *DBP*.

Data and samples from the six-month study follow-up visit were used for this analysis. No prior formal sample size calculation was performed; the first 500 patients recruited into the study who had both serum and urine available from their six-month visit were included; these visits occurred between April 2011 and September 2014. Participants were followed up from their six-month visit until death or the initiation of RRT. The last outcome data collection took place on 3^rd^ March 2016 and patients who had not reached a study end point were censored on this date.

### Laboratory analyses

Serum and urine were processed immediately after collection according to pre-defined standard operating procedures and stored at -80°C until analysis. Biochemistry results from the local clinical laboratory were obtained from tests performed in accordance with the current standard of care.

Serum ETP level was measured using a competitive enzyme-linked immunosorbent assay (ELISA) developed by Nordic Bioscience, Denmark. The monoclonal antibody employed in the ELISA detects the last 10 amino acids of the α3 chain of Col6 (^3168^’KPGVISVMGT’^3177^), but not the elongated peptide KPGVISVMGTA or truncated peptide KPGVISVMG; the technical details and characterization of the assay have previously been described [6].

Serum creatinine measurements were performed on a Roche Modular Analyser using a blank rated compensated Jaffe reaction, and eGFR was estimated using the creatinine-based CKD-EPI (Chronic Kidney Disease Epidemiology Collaboration) equation [18]. Urine ACR was measured using a Roche Hitachi 702 analyser.

C-reactive protein (CRP) was measured using the Full Range C-Reactive Protein Kit on a SPA^™^ automated PLUS turbidimeter (The Binding Site Group Ltd, Birmingham, UK). The normal range for CRP is between 0.1 and 9 mg/L, with 90 percent below 3 mg/L [19]. Serum kappa (κ) and lambda (λ) free light chain (FLC) concentrations were measured by nephelometry on a Dade-Behring BNTMII Analyser (Siemens AG, Erlangen, Germany) using particle enhanced high-specificity homogenous immunoassays (Freelite^™^; The Binding Site Group Ltd, Birmingham, UK). The normal reference ranges for serum FLC concentrations have been previously described as κ: 3.3–19.4 mg/L and λ: 5.7–26.3 mg/L, with the assay sensitivity being demonstrated as <1 mg/L. κ and λ FLC concentrations were combined to calculate the combined serum FLC (cFLC) concentrations.

### Statistical analyses

Statistical analyses were performed using IBM SPSS Statistics for Macintosh, Version 22 (Armonk, NY: IBM Corp, 2013). Baseline characteristics are described as a frequency and percentage for categorical variables, and as a mean and standard deviation or median and interquartile range (IQR) for normally and non-normally-distributed continuous variables respectively. As there were little missing data, complete case analysis was performed.

Serum ETP was analysed as both a continuous and a categorical variable by dividing the cohort into quartiles (Q1-4). Differences between ETP quartiles in baseline characteristics were assessed using Pearson’s chi-square for categorical variables, and ANOVA (parametric) or Kruskal-Wallis test (non-parametric) for continuous variables. ETP, eGFR, CRP, and cFLC were log transformed before scatter plots and Pearson’s correlation coefficients were used to assess for linear associations.

Kaplan-Meier survival analysis was performed for quartiles of ETP, and mean times to ESRD and death were estimated from the area under the survival curve. Cox proportional hazards regression was used to generate time-to-event data for progression to ESRD and death, and perform both univariable and multivariable analyses of the association between baseline characteristics and risk of ESRD and death. Known important confounding factors (eGFR and ACR) and any other variables with a *P*<0.1 on univariable analysis were included as candidate variables in the multivariable analysis, and a forward inclusion strategy was used to build final multivariable models. When variables were highly correlated, only the most clinically relevant was included as a candidate variable in the multivariable models: CKD-EPI eGFR was used and not Cystatin C; of the four blood pressure parameters (SBP, DBP, MAP, and PP), only the parameter most strongly correlated with the outcome of interest was used as a candidate variable, viz. MAP for progression to ESRD, and PP for mortality. Results are presented as a hazard ratio and 95% confidence interval (CI). The effect of adding ETP to a multivariable model was assessed by the change in chi-square with its associated *P* value (an increase in chi-square with a *P*<0.05 being taken as a significant improvement in model fit).

The primary outcomes of interest were 1) progression to ESRD and 2) death. ESRD was defined as the initiation of RRT (dialysis or renal transplantation) and was captured through the use of a local database of all patients who have commenced RRT. Patient mortality was captured through electronic patient records linked to the Office of National Statistics.

## Results

### Baseline characteristics

Of the 500 participants, two had an insufficient amount of serum available for analysis, so ETP levels were measured and analyses performed for 498 participants. No other participants were excluded from analysis. Baseline demographic, social, clinical, and laboratory data for the study participants, divided by serum ETP quartile, are presented in Table 1. The cohort was 61.4% male, median age was 64 years (IQR 50–76), and ethnicity was 72.3% white, 18.1% South Asian, 8.6% black, and 1.0% other. The primary renal diagnosis was ischaemic/hypertensive nephropathy in 28.4%, glomerulonephritis in 18.5%, diabetic kidney disease in 10.5%, polycystic kidney disease in 6.4%, and other/uncertain in 36.3%. Median eGFR was 26.5 mL/min/1.73 m^2^ (IQR 19.4–34.6), and median ACR was 32.1 mg/mmol (IQR 6.2–128.8). Median follow-up time, calculated using the reverse Kaplan-Meier method, was 37 months (range 0–58), and the shortest duration of event-free follow-up was 18 months; that is, any participants followed up for less than 18 months had reached a study end-point.

**Table 1:** Baseline demographic, social, and clinical characteristics of the cohort, divided into serum ETP quartiles.

	ETP Quartile				*P*	Completeness of data
	1	2	3	4		
	[6.8–16.8]	[16.9–23.0]	[23.1–30.0]	[30.1–77.5]		
n	125	124	125	124		
**Age (years)**	55.3 (14.8)	62.6 (17.5)	66.5 (15.7)	66.0 (14.7)	<0.0001	100%
**Sex (male)**	83 (66.4)	83 (66.9)	69 (55.2)	71 (57.3)	0.12	100%
**Ethnicity**					0.39	100%
**White**	92 (73.6)	86 (69.4)	96 (76.8)	86 (69.4)		
**South Asian**	16 (12.8)	24 (19.4)	23 (18.4)	27 (21.8)		
**Black**	15 (12.0)	13 (10.5)	5 (4.0)	10 (8.1)		
**Other**	2 (1.6)	1 (0.8)	1 (0.8)	1 (0.8)		
**Primary renal diagnosis**					<0.0001	91%
**Ischaemia/hypertension**	16 (14.0)	34 (31.5)	34 (29.8)	45 (37.8)		
**Diabetes**	4 (3.5)	8 (7.4)	8 (7.0)	28 (23.5)		
**Glomerulonephritis**	40 (35.1)	20 (18.5)	15 (13.2)	9 (7.6)		
**Polycystic kidney disease**	12 (10.5)	6 (5.6)	6 (5.3)	5 (4.2)		
**Other/uncertain**	42 (36.8)	40 (37.0)	51 (44.7)	32 (26.9)		
**Co-morbidities**						100%
**Cerebrovascular disease**	10 (8.0)	17 (13.7)	9 (7.2)	18 (14.5)	0.14	
**COPD**	14 (11.2	16 (12.9)	14 (11.2)	16 (12.9)	0.95	
**Diabetes mellitus**	36 (28.8)	33 (26.6)	47 (37.6)	67 (54.0)	<0.0001	
**Ischaemic heart disease**	20 (16.0)	25 (20.2)	34 (27.2)	33 (26.6)	0.10	
**Malignancy**	17 (13.6)	19 (15.3)	20 (16.0)	15 (12.1)	0.82	
**Peripheral vascular disease**	5 (4.0)	19 (15.3)	16 (12.8)	11 (8.9)	0.019	
**Age-adjusted CCI (score ≥5)**	41 (32.8)	67 (54.0)	80 (64.0)	90 (72.6)	<0.0001	100%
**Smoking status (current smoker)**	26 (20.8)	13 (10.5)	15 (12.0)	13 (10.5)	0.048	100%
**IMD Score**	24.5 (15.7–43.2)	31.7 (18.8–46.1)	28.0 (15.3–43.7)	30.8 (19.7–47.2)	0.18	100%
**Body mass index (kg/m^2^)**	28.6 (5.5)	28.5 (5.8)	29.8 (7.1)	32.1 (8.2)	<0.0001	99%
**Systolic blood pressure (mmHg)**	121 (16)	127 (21)	130 (22)	132 (22)	<0.0001	100%
**Diastolic blood pressure (mmHg)**	78 (11)	76 (12)	74 (11)	72 (11)	0.001	100%
**Mean arterial pressure (mmHg)**	92 (11)	93 (13)	93 (12)	92 (12)	0.91	100%
**Pulse pressure (mmHg)**	39 (33–48)	48 (47–63)	53 (40–67)	56 (44–72)	<0.0001	100%
**Serum creatinine (μmol/L)**	151 (47.0)	203 (51.2)	238 (66.5)	283 (96.0)	<0.0001	99%
**Cystatin C (mg/L)**	1.69 (0.43)	2.34 (0.42)	2.86 (0.48)	3.34 (0.63)	<0.0001	94%
**CKD-EPI eGFR (mL/min/1.73m^2^)**	41 (33–54)	28 (23–33)	22 (18–27)	18 (14–24)	<0.0001	99%
**Albumin creatinine ratio (mg/mmol)**	28.0 (4.2–124.4)	30.0 (5.7–115.3)	27.1 (6.5–129.7)	45.4 (9.1–154.7)	0.14	98%
**C-reactive protein (mg/L)**	1.8 (0.8–4.6)	2.8 (1.2–5.4)	3.5 (1.9–7.0)	4.4 (2.0–11.5)	<0.0001	94%
**Serum cFLC (mg/L)**	49.9 (35.3–62.0)	69.8 (55.7–93.0)	88.8 (71.0–110.8)	115.4 (89.7–157.7)	<0.0001	94%
**Serum ETP (ng/mL)**	13.7 (11.6–15.2)	20.0 (18.4–21.5)	25.9 (24.5–28.2)	36.4 (32.3–42.2)	<0.0001	100%

Categorical variables are expressed as number (%), and continuous variables as mean (SD) or median (IQR). ETP = endotrophin; COPD = chronic obstructive pulmonary disease; CCI = Charlson’s comorbidity index; CKD-EPI = Chronic Kidney Disease Epidemiology Collaboration; eGFR = estimated glomerular filtration rate; cFLC = combined free light chains.

**Fig 1:**
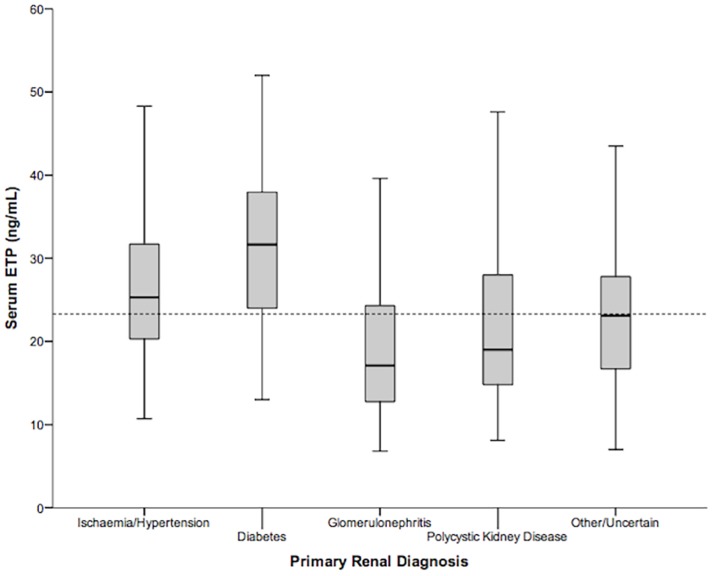
Boxplots showing serum ETP levels by primary renal diagnosis. The interrupted line represents median ETP level for the whole cohort. ETP = endotrophin.

Median ETP level was 23.1 ng/mL (IQR 16.8–30.1). Participants in each ETP quartile were similar in terms of gender, ethnicity, and socioeconomic status, as shown in Table 1. However, patients in higher quartiles were older, more likely to have diabetes mellitus (DM), and had more comorbidity (as assessed by an age-adjusted CCI of ≥5). Patients in higher quartiles also had a higher BMI, higher SBP, and lower DBP. Patients in higher quartiles were more likely to have ischaemic/hypertensive nephropathy or diabetic kidney disease as their primary renal disease and less likely to have glomerulonephritis, and also had worse renal function (lower eGFR and higher cystatin C), higher CRP, and higher serum cFLC. There was no significant difference between quartiles in MAP and urinary ACR. ETP levels by primary renal disease are shown in Fig 1.

**Fig 2:**
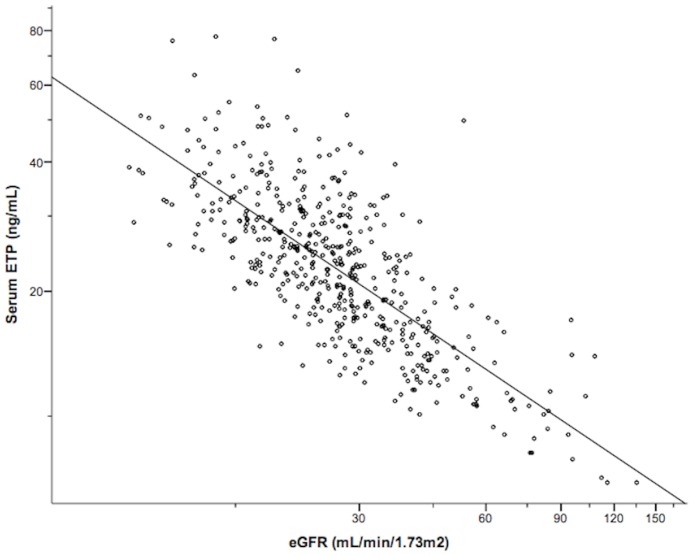
Association between renal function and serum ETP level. Scatter plot of ETP by CKD-EPI eGFR (logarithmic scales), R^2^ = 0.547.

**Fig 3:**
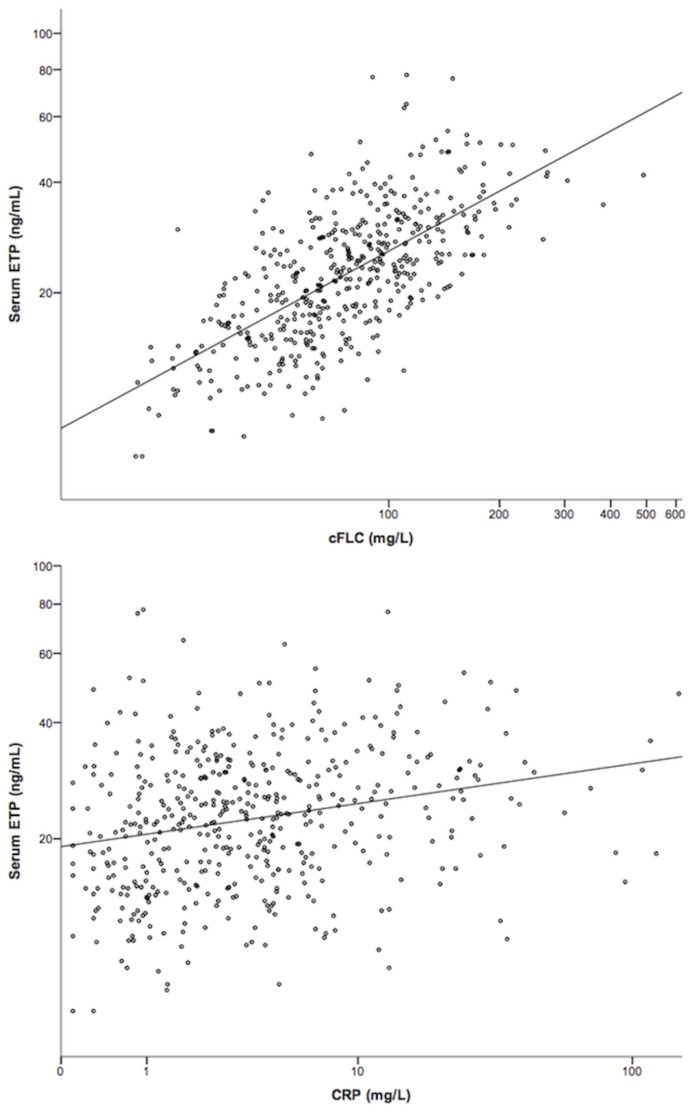
Scatter plots showing the association between ETP and markers of systemic inflammation. a) ETP by cFLC, R^2^ = 0.445; b) ETP by CRP, R^2^ = 0.057. All scales are logarithmic. ETP = endotrophin; cFLC = combined serum free light chains; CRP = C-reactive protein.

After log transformation of both variables, there was a strong linear association between serum ETP level and eGFR (Pearson’s correlation coefficient r = -0.74, *P*<0.0001), indicating that ETP level increases with decreasing eGFR as shown in Fig 2. ETP level also showed a strong positive correlation with cFLC (r = 0.67, *P*<0.0001) and a weak correlation with CRP (r = 0.25, *P*<0.0001), as illustrated in Fig 3. There was no significant correlation between ETP and ACR (r = 0.131, *P* = 0.07).

### Progression to ESRD

One hundred and four (20.9%) participants progressed to ESRD, and the estimated mean time to ESRD was 48.7 (95% CI 47.0–50.3) months. Univariable Cox regression analysis of baseline characteristics and their hazard ratio for progression to ESRD is shown in Table 2. ETP level when analysed as both a continuous variable and by quartiles, was significantly associated with progression to ESRD. Hazard ratios for an increase in ETP of 5, 10, and 20 ng/mL were 1.34 (1.26–1.42), 1.79 (1.59–2.02), and 3.20 (2.51–4.07), respectively (all *P*<0.0001), and participants in the highest quartile had a hazard ratio for progression to ESRD 11-fold that of the lowest quartile. The number and percentage of patients reaching ESRD in each ETP quartile were 7 (5.6%), 21 (16.9%), 29 (23.2%), and 47 (37.9%) in Q1-Q4, respectively.

**Table 2:** Univariable Cox regression analysis of variables associated with progression to ESRD and death.

	Progression to ESRD		Death	
	Hazard Ratio (95% CI)	*P*	Hazard Ratio (95% CI)	*P*
**Age (years)**^a^	0.90 (0.81–1.01)	0.08	2.16 (1.72–2.72)	<0.0001
**Sex**		0.61		0.33
**Female**	1		1	
**Male**	0.90 (0.61–1.34)		1.29 (0.77–2.15)	
**Ethnicity**		0.052		0.80
**White**	1		1	
**South Asian**	1.84 (1.17–2.90)		0.80 (0.39–1.61)	
**Black**	1.56 (0.84–2.89)		0.65 (0.24–1.80)	
**Other**	0.90 (0.13–6.52)		1.13 (0.16–8.21)	
**Primary renal diagnosis**		0.001		0.012
**Ischaemia/hypertension**	1		1	
**Diabetes**	2.32 (1.27–4.26)		1.23 (0.57–2.66)	
**Glomerulonephritis**	0.98 (0.53–1.79)		0.12 (0.03–0.49)	
**Polycystic kidney disease**	2.21 (1.13–4.32)		0.36 (0.09–1.53)	
**Uncertain/other**	0.84 (0.49–1.44)		0.62 (0.34–1.14)	
**Co-morbidities**				
**Cerebrovascular disease**	0.93 (0.48–1.78)	0.82	1.92 (1.03–3.59)	0.041
**COPD**	0.52 (0.24–1.11)	0.09	1.74 (0.95–3.19)	0.07
**Diabetes mellitus**	0.98 (0.66–1.47)	0.93	2.27 (1.40–3.69)	0.001
**Ischaemic heart disease**	0.98 (0.62–1.58)	0.95	2.69 (1.65–4.39)	<0.0001
**Malignancy**	0.47 (0.23–0.97)	0.040	1.67 (0.94–2.97)	0.08
**Peripheral vascular disease**	1.02 (0.55–1.91)	0.94	1.90 (1.02–3.56)	0.044
**Age-adjusted CCI (score ≥5)**	0.68 (0.46–1.00)	0.049	17.79 (5.59–56.65)	<0.0001
**Smoking status**		0.96		0.18
**Never smoked**	1		1	
**Ex smoker**	1.06 (0.70–1.60)		1.56 (0.92–2.63)	
**Current smoker**	1.08 (0.60–1.94)		0.94 (0.40–2.19)	
**IMD Score**^a^	1.08 (0.96–1.21)	0.19	1.09 (0.94–1.25)	0.25
**Body mass index (kg/m^2^)**^b^	1.06 (0.92–1.22)	0.45	0.99 (0.82–1.19)	0.92
**Systolic blood pressure (mmHg)**^a^	1.19 (1.10–1.29)	<0.0001	1.23 (1.12–1.36)	<0.0001
**Diastolic blood pressure (mmHg)**^b^	1.14 (1.06–1.24)	0.001	0.90 (0.80–1.00)	0.05
**Mean arterial pressure (mmHg)**^b^	1.17 (1.09–1.26)	<0.0001	1.06 (0.96–1.17)	0.27
**Pulse pressure (mmHg)**	1.01 (1.00–1.02)	0.017	1.03 (1.02–1.04)	<0.0001
**Serum creatinine (μmol/L)**^c^	1.27 (1.22–1.32)	<0.0001	1.09 (1.02–1.16)	0.010
**Cystatin C (mg/L)**	3.03 (2.38–3.85)	<0.0001	2.38 (1.75–3.24)	<0.0001
**CKD-EPI eGFR (mL/min/1.73m^2^)**^a^	0.26 (0.19–0.35)	<0.0001	0.57 (0.43–0.76)	<0.0001
**Albumin creatinine ratio (mg/mmol)**^a^	1.03 (1.03–1.04)	<0.0001	1.01 (0.99–1.03)	0.36
**C-reactive protein (mg/L)**^b^	0.98 (0.91–1.06)	0.62	1.02 (0.95–1.09)	0.57
**Serum cFLC (mg/L)**^c^	1.23 (1.19–1.29)	<0.0001	1.19 (1.12–1.27)	<0.0001
**ETP (ng/mL)**^a^	1.79 (1.59–2.02)	<0.0001	1.60 (1.35–1.89)	<0.0001
**Q1**	1	<0.0001	1	<0.0001
**Q2**	3.36 (1.43–7.90)		3.30 (1.06–10.24)	
**Q3**	4.90 (2.15–11.19)		5.88 (2.01–17.20)	
**Q4**	11.05 (4.98–24.52)		12.14 (4.26–34.54)	

ESRD = end-stage renal disease; CI = confidence interval; COPD = chronic obstructive pulmonary disease; CCI = Charlson’s comorbidity index; CKD-EPI = Chronic Kidney Disease Epidemiology Collaboration; eGFR = estimated glomerular filtration rate; cFLC = combined free light chains; ETP = endotrophin; Q = quartile.

^a^Analysed in 10-unit increases.

^b^Analysed in 5-unit increases.

^c^Analysed in 20-unit increases.

Other baseline characteristics significantly associated with increased risk of progression to ESRD by univariable analysis included South Asian ethnicity, a primary renal diagnosis of diabetic nephropathy or polycystic kidney disease, an age-adjusted CCI score <5, absence of malignancy, higher SBP, higher DBP, higher MAP, higher cystatin C, lower CKD-EPI eGFR, increasing urine ACR, and higher serum cFLC.

After adjusting for eGFR and ACR, ETP was no longer independently associated with progression to ESRD (HR 1.19 [0.98–1.45], *P* = 0.08). Cox p4 follows:-, ows—Quartile ing ESRD in each quartile was as follows: quartile 1 5.6%, Q2riable and by quartiles, is signifiMultivariable Cox regression using a forward selection procedure produced a final model containing eGFR, ACR, age, MAP, cFLC, and a renal diagnosis of polycystic kidney disease, as shown in Table 3.

**Table 3:** Final multivariable Cox regression model for progression to ESRD.

Variable	Hazard Ratio (95% CI)	*P*
**CKD-EPI eGFR**^a^	0.22 (0.15–0.32)	<0.0001
**ACR**^a^	1.04 (1.03–1.05)	<0.0001
**Age**^a^	0.72 (0.62–0.83)	<0.0001
**Mean Arterial Pressure**^b^	1.15 (1.06–1.25)	<0.0001
**cFLC**^c^	1.14 (1.08–1.20)	<0.0001
**Polycystic kidney disease**	3.34 (1.80–6.18)	<0.0001

CI = confidence interval; CKD-EPI = Chronic Kidney Disease Epidemiology Collaboration; eGFR = estimated glomerular filtration rate; ACR = albumin creatinine ratio; cFLC = combined free light chains.

^a^Analysed in 10-unit increases.

^b^Analysed in 5-unit increases.

^c^Analysed in 20-unit increases.

### Mortality

**Fig 4:**
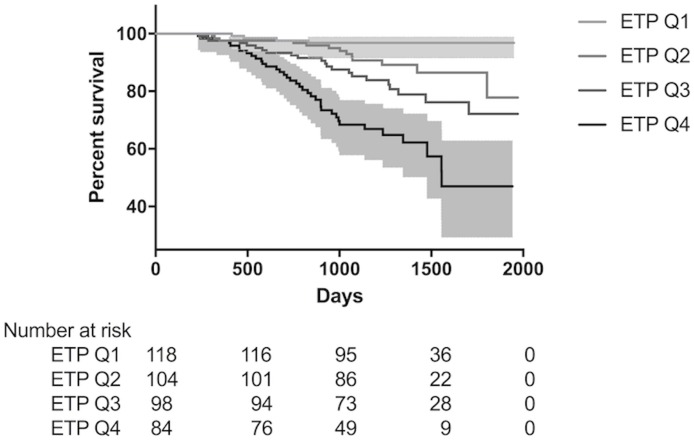
Kaplan-Meier plot showing survival by ETP quartile. 95% CI for quartiles 1 and 4 are represented by the grey areas. ETP = endotrophin.

Sixty-six (13.3%) participants died, and the estimated mean survival time was 52.0 (95% CI 50.6–53.5) months. Univariable analysis of baseline characteristics and their hazard ratio for risk of death is shown in Table 2. ETP level was significantly associated with mortality when analysed as both a continuous variable and by quartiles. Hazard ratios for a 5, 10, and 20 ng/mL increase in ETP were 1.26 (1.16–1.37), 1.60 (1.35–1.89), and 2.55 (1.83–3.57), respectively (all *P*<0.0001), and patients in the highest quartile had a 12-fold increased risk of death relative to the lowest quartile. The number and percentage of participants in each ETP quartile who died were 4 (3.2%), 12 (9.7%), 20 (16.0%), and 30 (24.2%), and estimated mean survival times were 56 (54–57), 54 (51–56), 50 (47–53), and 43 (39–47) months, in Q1-4 respectively. A Kaplan-Meier plot showing survival by ETP quartile is shown in Fig 4.

Other baseline characteristics significantly associated with an increased risk of mortality were increasing age, DM, cardiovascular disease (ischaemic heart disease [IHD]), cerebrovascular disease [CVD], peripheral vascular disease [PVD]), an age-adjusted CCI ≥5, higher SBP, increased PP, increasing cystatin C, lower eGFR, and higher serum cFLC.

On multivariable analysis, after adjusting for age, eGFR, ACR, co-morbidities, PP and cFLC, ETP significantly improved model fit (*P* = 0.030) and remained significantly associated with mortality (HR 1.51 [1.07–2.12], *P* = 0.019). A forward selection procedure produced a final multivariable model for mortality containing age, IHD, ACR, and ETP, as shown in Table 4. These analyses indicate that ETP remained independently significantly associated with mortality after adjustment for confounding factors.

**Table 4:** Final multivariable Cox regression model for mortality.

Variable	Hazard Ratio (95% CI)	*P*
**Age**^a^	2.20 (1.68–2.87)	<0.0001
**Ischaemic heart disease**	1.78 (1.04–3.04)	0.035
**ACR**^a^	1.03 (1.01–1.05)	0.004
**ETP**^a^	1.69 (1.32–2.17)	<0.0001

ACR = albumin creatinine ratio; ETP = endotrophin.

^a^Analysed in 10-unit increases.

Of the 66 participants who died, the cause of death was available for 58 and was CVD in 17 (29%), malignancy in 16 (28%), infection in 9 (15%), and other in 16 (28%). Median (IQR) serum ETP was 30.4 (20.5–35.8) for CVD, 24.5 (19.3–29.6) for malignancy, 28.3 (24.9–31.4) for infection, and 31.4 (25.2–37.0) for other, and was not significantly different when analysed by cause of death (*P* = 0.12).

## Discussion

Identifying the dominant biological processes associated with worse outcomes in patients with CKD will facilitate our understanding of pathophysiology and identify targets that may lead to improved outcomes, both through stratification and intervention. Tissue fibrosis and remodelling have been implicated in both the progression of CKD and the increased mortality risk associated with CKD. However, little is known about the associations between systemic fibrosis and increased clinical risk in human kidney disease. We analysed serum ETP, a marker of Col6 formation, in a cohort study of high-risk CKD and found an independent relationship between ETP level and mortality.

There was a significant inverse correlation between eGFR and serum ETP levels, for which there are several possible explanations. Although the molecular weight of ETP is not known, the correlation may reflect a reduction in renal clearance of ETP as kidney function declines. It may also reflect increased production of fibrotic tissue, and therefore Col-6 and ETP generation, in patients with CKD: renal fibrosis is known to be a common pathological pathway in CKD and may therefore be a contributing source of ETP, but the elevated levels are also likely to reflect a greater systemic fibrotic burden in patients with more advanced CKD. Our data do not allow us to determine the relative contributions of these potential explanations for the correlation described. We also recognise the possibility that the assay detects peptides of different length that all terminate with the 10 amino acid sequence targeted by the ELISA, among which is ETP.

Higher ETP levels were associated with both an increased risk of progression to ESRD and an increased risk of death. In multivariable models including conventional risk factors, ETP was no longer associated with an increased risk of progression to ESRD. However, the significant association between ETP and mortality remained, which may suggest a novel contribution of systemic fibrosis to the increased mortality seen in CKD. Further, ETP was selected into the final multivariable model for mortality rather than eGFR (one of the most important prognostic factors for mortality in CKD), indicating superior predictive power in this cohort.

There are several possible reasons for the significant independent association between serum ETP level and increased mortality risk. In addition to reflecting increased systemic fibrosis and therefore increased mortality risk, both Col6 and ETP have known adverse effects which may be associated with reduced survival. For example, Col6 has a major role in platelet adhesion, which is intimately involved in both atherosclerosis and microvascular pathology. Col6 binds platelets both directly and via von Willebrand factor (vWF), and of the multiple subendothelial collagens to which vWF binds, Col6 appears to be especially important [20, 21][22]. Col6 also appears to have deleterious effects in the myocardium: Col6 deletion in knockout mice is associated with improved cardiac function, structure and remodelling after myocardial infarction, and in humans with hypertrophic cardiomyopathy, increased levels of Col6 positively correlate with cardiac interstitial fibrosis and dysfunction [23, 24]. The hypothesis that increased Col6 expression may be linked with an increased risk of cardiovascular death would have been supported had our data revealed a higher serum ETP level in those who died from CVD. Although this was not the case, the risk of a type II error is significant given the small numbers in each category of cause of death.

The adverse effects of ETP that might explain its association with increased mortality include its pivotal role in shaping a metabolically unfavourable microenvironment in adipose tissue, where it triggers fibrosis and inflammation and ultimately results in systemic elevation of pro-inflammatory cytokines, insulin resistance, and the metabolic syndrome [25]. We have recently demonstrated that elevated ETP levels predict response to insulin sensitizers in patients with type 2 DM [26]. Further, ETP has been shown to play a role in promoting tumour growth and metastasis [3, 4, 27].

It is also possible that the relationship between Col6 expression, ETP level, and increased mortality is not causal. Rather, ETP may be a confounder, increased in response to factors that in themselves increase mortality risk. For example, increased ECM deposition may be a consequence of pathogenic triggers such as hypertension and increased vascular sheer stress; notably, the presence of Col6 has been demonstrated in the thickened intima of atherosclerotic lesions in humans [28]. Hypertension, DM, and myocardial infarction are also associated with increased cardiac Col6 expression [29, 30]. However, the role of Col6 in the myocardial fibrosis seen in patients with CKD is unknown.

The strengths of this study include it being a large prospective cohort, with detailed bio-clinical phenotyping incorporating multiple risk factors for adverse outcomes. The study’s limitations include it being single-centre, with no validation cohort, and the lack of experimental data to explore the mechanisms underlying the association between ETP and mortality. However, this work provides the basis for confirmatory and mechanistic studies in this important clinical area.

In conclusion, this study shows that serum ETP levels are independently associated with mortality risk in CKD after adjustment for confounding factors.
